# Early Attraction in Temporally Controlled Sight Reading of Music

**DOI:** 10.16910/jemr.11.2.3

**Published:** 2018-04-10

**Authors:** Erkki Huovinen, Anna-Kaisa Ylitalo, Marjaana Puurtinen

**Affiliations:** Royal College of Music in Stockholm,, Sweden; University of Jyväskylä, Finland; University of Turku, Finland; [www.kmh.se, www.jyu.fi]

**Keywords:** Eye tracking, Eye-hand span, Eye-time span, Meter, Music reading, Parafoveal processing, Perceptual span, Psychology of Music, Saccadic control, Tempo

## Abstract

A music reader has to “look ahead” from the notes currently being played—this has usually been called the Eye-Hand Span. Given the restrictions on processing time due to tempo and meter, the Early Attraction Hypothesis suggests that sight readers are likely to locally increase the span of looking ahead in the face of complex upcoming symbols (or symbol relationships). We argue that such stimulus-driven effects on looking ahead are best studied using a measure of Eye-Time Span (ETS) which redefines looking ahead as the metrical distance between the position of a fixation in the score and another position that corresponds to the point of metrical time at fixation onset. In two experiments of temporally controlled sight reading, musicians read simple stepwise melodies that were interspersed with larger intervallic skips, supposed to create points of higher melodic complexity (and visual salience) at the notes following the skips. The results support both Early Attraction (lengthening of looking ahead) and Distant Attraction (lengthening of incoming saccades) in the face of relative melodic complexity. Notably, such effects also occurred on the notes preceding the nominally complex ones. The results suggest that saccadic control in music reading depends on temporal restrictions as well as on local variations in stimulus complexity.

## Introduction

Reading musical notation shares some commonalities with reading linguistic texts, including a linear progression from left to right and the possibility of involving auditory imagery even in silent reading. There are nevertheless at least two important differences between these two reading domains that make it impossible to learn about the processes of music reading directly from studies of language reading. First, music does not rely on a lexicon and referential semantics in the sense that language does, and hence it does not have fixed “words” in the linguistic sense. However, morphological units may arise through grouping mechanisms that in the written domain can be traced down to pitch structure and temporal structure (e.g., groupings of notes separated by larger differences in pitch or time), articulation and phrasing markings (e.g., slurs, curved lines indicating that a group of notes is to be articulated together), and orthographic conventions and decisions (e.g., beaming several eighth notes together with a thick vertical line connecting the note stems: 
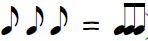
). It is common understanding that musical notation can be easy or difficult to read due to compositional decisions made on all of these levels. Therefore, reading musical notation often does resemble reading a foreign language text in the sense that some of its smallest structural units can be more difficult than others to sight read (i.e., to read at first sight) even for competent musicians, and that such details may have to be deciphered and practiced before they can be fluently “read out loud.” 

The second crucial difference between reading music and reading text is due to the fact that musical notation is most often (but not exclusively) understood as a guide to instrumental performance, and such performances are typically (but not exclusively) expected to follow rather strict temporal constraints. In musical traditions relying on the Western notation system, musicians typically synchronize their performances by keeping to a common tempo and meter—a cyclically recurring, hierarchical scheme of “strong” and “weak” beats that is regulated in the notation by a time signature (e.g., 4/4, meaning four quarter notes within a bar: 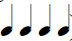). A solo performer, reading music from the score, must likewise more or less hold on to such metrical constraints and is not free to stop and ponder a more interesting or difficult passage in the notation in the way that a language reader most often can. Even outside of performance contexts, the reader of musical notation is arguably required to understand the “meaning” of the written music in terms of its temporal and metrical constraints.

The present study sets out to explain how music readers handle local musical complexities in sight reading, while conforming to the constraints of the musical meter in a set tempo. To the extent that the sight reader wishes to maintain a constant flow of music and thus only has a limited amount of time available for reading each metrical unit (such as a bar, separated by two vertical bar lines), how will she regulate her eye movements to cope with more challenging musical details that may crop up in the notation? From previous studies, we know that comparatively difficult elements in the notation—such as incongruent endings (1) or notes appearing after larger intervallic skips in an otherwise stepwise melodic context (2)—may require relatively more total fixation time than other elements to be accurately processed in a sight-reading performance (3). Conceivably, and irrespective of fixation time allocated to them, such details might often require more time for planning the motor sequence—that is, more time between the first fixation onset to the target, and the moment when a corresponding sound has to be produced on a musical instrument. In a temporally regulated context, such extra time has to be compensated for by decreasing the time spent on other elements (4, 5). If the music progresses at a more or less uniform tempo, any upcoming difficulties have to be spotted well in advance so that the performer has enough time—despite the locally heavier processing load—to decipher the difficult symbols and prepare a motor sequence for executing the notes on an instrument. Such spotting in advance, however, would require being alert to what musical information enters the parafoveal area, outside of the currently fixated note symbols.

### Early Attraction and Distant Attraction in Music reading

Despite the differences between the domains of reading music and reading text, it is instructive to approach the issue by briefly reviewing some of the findings concerning parafoveal processing in text reading. The influences of words on eye movements in reading are typically broken down to questions of where to move the eyes and when to move them (6, 7). The general understanding is that information obtained foveally—from the fixated word—largely determines when to move the eyes, whereas parafoveal information—understood in this context as information concerning the next, non-fixated word(s)—is responsible for where to move them. Considering the kinds of properties relevant to foveal and parafoveal processing, it is also possible to say that cognitive/linguistic properties of a word, such as frequency and predictability, mostly determine the duration of a fixation on the word before moving on, while visual properties, such as word length and orthography, have the largest effect on landing position of the next fixation. (For reviews, see (8, 6).) In particular, Hyönä (9) found that irregular strings of letters in the beginning of a word tend to attract, or “pull” (10), the first fixation closer to the beginning of the word, and even to the space prior to the word (in comparison to the so-called preferred viewing location just left of the center of the word). Likewise, White and Liversedge (11, 12, 13) demonstrated that for misspelled words, the landing positions for incoming saccades tend to be nearer the beginning of the word. There is also some evidence that low-frequency words may attract eye movement (14). However, to set the stage for our musical study, we need not concern ourselves with the distinction between orthographic and lexical information. As should be clear from above, any such distinctions would have to be made on independent grounds in the case of music. What we do get from these results is the following robust, overall picture: Upcoming symbols that are in some sense “irregular” or less typical—and hence potentially more difficult—may guide saccadic planning even before they are fixated.

**Fig 1.**
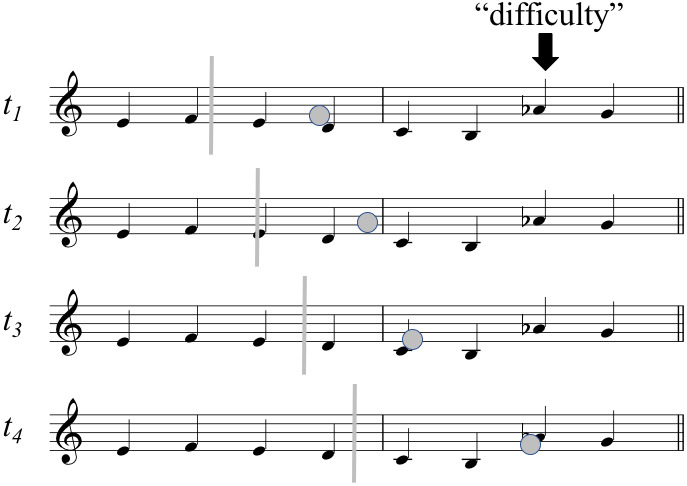
A schematic example of our Early Attraction hypothesis in sight reading: The gray cursor marks the metrical time sliding continuously across the score; The circle marks the reader’s concurrent fixation. At moment *t*_*4*_, a difficulty attracts the reader’s eyes, stretching the span between the fixation and the cursor.

Let us now return to our musical problem situation. In simple sight-reading tasks, such as ones incorporating only quarter-notes (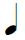) on a diatonic scale (e.g., the collection of notes on the white keys of the piano), it is typical that the sight reader has time to fixate most of the successive notes (e.g., (15)); thus, making conclusions about “where in the word” the fixations land is not enough to diagnose the process. As already indicated above, it would instead be reasonable to expect that any salient difficulties spotted in the parafovea would affect the “when” question—the timing of the saccade launched ahead. In other words, it could be expected that salient difficulties in a musical score attract first fixations relatively early on in the course of the musical performance, helping the reader allocate enough of the limited processing time to the difficulties. This could be called the Early Attraction Hypothesis (cf. the merely spatial attraction hypothesis that could be used to account for the above results in language reading; see 9). If we imagine the passing of the metrical time of music as a cursor gradually sliding across the score, touching the note symbols as they are performed, the reader’s fixations would always tend to be a bit ahead of the cursor. Figure 1 gives a simplified example of the expected effect by depicting four successive moments *t*_*1*_*–t*_*4*_ in an imagined sight-reading performance. Until *t*_*3*_, the reader’s eyes are more or less similarly ahead of the cursor. In relation to such a process, Early Attraction would postulate a processing difficulty to be reflected by a fixation landing further to the right of the sliding cursor, thus increasing the time for identifying the symbol in question and for planning a suitable motor action. In our hypothetical example, the sudden leap of the melody to a higher note, together with the accidental sign prefixed to it (further modifying the pitch by a semitone), constitutes a relative “difficulty” that attracts the reader’s eyes at *t*_*4*_, in this case even causing the reader to skip fixating the preceding note.

Early Attraction, as here conceived, is akin to the so-called (inverted) parafoveal-on-foveal effects observed in text reading, but is not tantamount to any one of them, as standardly defined. Parafoveal-on-foveal effects often involve the increase of foveal fixation durations in the face of parafoveal processing difficulties (e.g., due to orthography: (16, 17, 18)), but also inverted effects—shorter focal fixations due to parafoveal difficulties—have been shown in some experiments (14, 19, 21). In the latter case, researchers might speak of an “early saccade toward the location of the parafoveal difficulty” (20), p. 654, “earliness” being understood with reference to the previous fixation onset. Another kind of inverted parafoveal-on-foveal effect is skipping the target word when the parafoveal word presents some irregularities—as if the irregularity would operate as a “magnet” attracting the eyes (14). Our Early Attraction Hypothesis would be compatible with both of these inverted effects, but it says nothing directly about shortened fixation durations or skipping. Instead, it is a higher-level assumption about when visual information would need to be gathered in relation to the passing of metrical time (see below). As such, it corresponds to the “magnet account” that Hyönä and Bertram (14) use to cover both of the inverted effects mentioned above. But whereas Hyönä and Bertram acknowledge that their account “makes a counterintuitive prediction in claiming that parafoveal processing difficulty should lead to shorter processing times for the foveal word” (14), p. 124, we submit that, in temporally regulated music-reading contexts, such a phenomenon would be far from counterintuitive. In cases such as the one depicted in our imaginary example above, an early glance to a “difficult” target could be achieved by terminating a prior fixation earlier than otherwise, but it might also be a matter of skipping some fixation(s) that might have otherwise occurred. At the present state of research, the difference seems immaterial. The variability in music readers’ eye-movement processes and the abundance of big and small irregularities in musical scores do not suggest analyzing such “earliness” in relation to some “regular” pattern of fixation locations or fixation times. The effect, if one exists, is better identified on the level of the music reader’s management of time resources in general—as being early when needed.

Given that musical notes not only take time to be performed but also occupy graphical space in the visual score, Early Attraction would generally imply reacting to such difficulties over a larger spatial span as well. If so, we might additionally hypothesize that the saccades landing on difficult symbols might be longer than average. This is a separate assumption that will be called the Distant Attraction Hypothesis. Supposing Early Attraction to be attested, Distant Attraction would represent the simplest way it could take place: The reader would react to an upcoming difficult target by directing a single, longer saccade toward it well before reaching that point in the music. This would indeed be the case in the schematic example of Fig. 1, supposing that the four successive moments depict successive fixations: The horizontal distance between fixations lengthens between moments *t*_*3*_ and *t*_*4*_. In text reading, an analogous effect does not seem to occur, however: Hyönä (9) reported that the distance to the launch site (the location of the previous fixation) is not influenced by the type of target word for a saccade. In fact, studies in reading Chinese suggest quite the opposite to what we would be expecting: longer saccades being associated with less complex characters (22) and with higher-frequency characters and words (22, 23). By comparison to music reading, the absence of Distant Attraction might not here be utterly surprising: The language reader is not obliged to have finished the whole sentence or the whole paragraph in a given total time, and hence there is no crucial benefit for launching longer saccades to upcoming difficulties early on in the process. Here, any additional processing time needed for a difficult target can remain a local temporal extension, rather than requiring a balancing act somewhere else in the process. In the case of temporally regulated musical sight reading, however, such balancing acts become a necessity as soon as one of the symbols requires extra processing time. The Early Attraction Hypothesis suggests that this may happen as a prior adjustment to parafoveally presented information, and the Distant Attraction Hypothesis further suggests that the mechanism for being early would be in terms of single, longer jumps ahead.

The joint hypotheses concerning Early and Distant Attraction mean that saccadic programming may be, in part, locally influenced by musical detail in the notated score—particularly by the salient and/or difficult elements in it. We will discuss such elements using the concept of complexity. Generally, we know that the overall level of complexity in the sight-reading stimulus may affect which skills of the reader are important for successful performance (24), but complexity may also vary within the stimulus, as already implied. Instead of giving a formal definition, we will treat local complexity as a heuristic notion that is always relative to the notated musical surroundings. Embedded amidst an otherwise simple diatonic melodic texture, a sudden note with an accidental sign (i.e., sharp [#] or flat [b], raising or lowering the note by a semitone) would tend to mark a deviation from the expected. Here, complexity could be seen as intrinsic to the compound symbol itself, but notice that local musical complexities may also be relational, not reducible to the individual elements. Consider again the melodic example of Fig. 1. For the first six notes, it only uses the white keys of the piano, proceeding stepwise on the diatonic scale (as indicated by note heads in adjacent positions on the lines and in the spaces of the musical staff). Such melodic movement might be decoded and executed simply as a series of “up” and “down” commands, but any larger intervallic skip to a higher or lower note might require identifying the note after the skip by its name (or by its position on the keyboard) in order for it to be correctly performed (2). In this cognitive sense, the “difficult” note of Fig. 1 could indeed represent greater relational complexity than its immediate surroundings, even if the accidental sign was removed from it.

We suggest that even simple musical notation thus involves variations in structural complexity—and hence, constantly shifting levels of processing load—that may affect skilled musicians’ sight-reading performance. Notice, too, that points of musico-syntactical complexity can often be expected to correspond to points of visual saliency in a musical score. According to music-theoretical lore, an intervallic skip such as that in Fig. 1 is likely to be heard as a melodic grouping boundary in the auditory domain (25), p. 46, but it also brings about a visual grouping boundary in the vertical dimension of the score: The last two notes of the example seem to form a visual group of their own, beginning on a note that is thus salient in its surroundings. From research on picture perception we know that highly salient objects attract fixations earlier than less conspicuous ones (when the task requires encoding the whole picture; (26, 27)); in music reading, we may well suppose a similar mechanism to function as an aid to saccadic programming, which would facilitate allocating the limited processing time to where it is most sorely needed.

### The Eye-Hand Span and the Eye-Time Span

In previous research on eye movements in music reading, the central concept used in discussions of temporal control has been the Eye-Hand Span. In proposing the Early and Distant Attraction Hypotheses, we are, in effect, predicting local increases of the Eye-Hand Span due to musico-visually complex features of the notated musical stimulus. That is, we suggest that local, upcoming complexities in the score might lead the sight-reader to “look farther ahead” than usual from the notes currently being played. In studies of the Eye-Hand Span, such a possibility has not been investigated before. To see why, we need to take a closer look at how the span has been defined. In studies on music reading, the Eye-Hand Span has generally been understood as the “distance between production and perception” (28), p. 161. Operationalizations for this concept have varied. It has been defined in terms of the number of notes (29, 30, 31) or beats (4, 32, 33), or with regard to spatial distance (34) or absolute time (29, 30, 33). Studies suggest that more experienced music readers apply larger Eye-Hand Spans than less experienced ones, when the span is calculated in terms of spatial distance (32, 34), beats (4), or the number of notes (31). In terms of absolute time, Furneaux and Land (29) reported an average Eye-Hand Span to lie around 1 s for both amateur and professional musicians, and it is worth noting that even experienced sight readers may not, in fact, use spans as large as sometimes believed (32). Most studies on the Eye-Hand Span have not externally controlled the performance tempo (exceptions being (4, 29, 33)), and thus surprisingly little is still known about the effects of regulated tempo on looking ahead in music reading.

**Fig 2.**
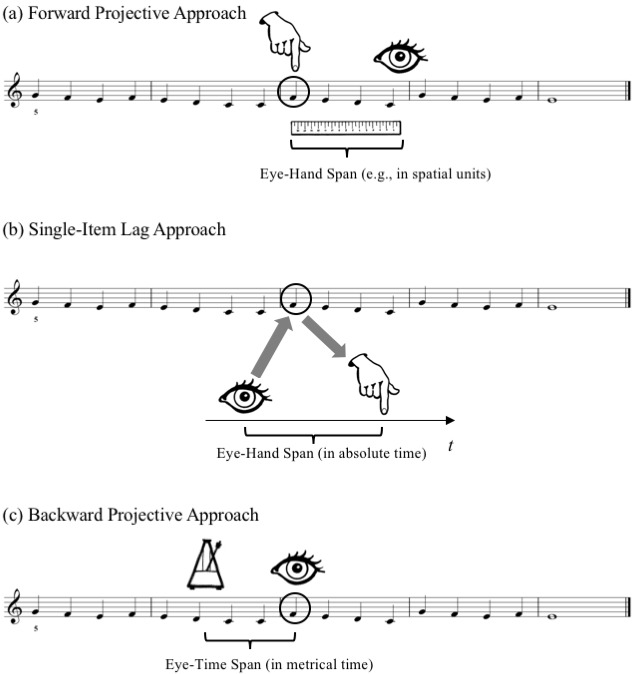
Three approaches to span measurement: (a) The Forward Projective Approach and (b) the Single-Item Lag Approach, resulting in two variants of the Eye-Hand Span; (c) The Backward Projective Approach, resulting in the Eye-Time Span.

When the span is measured “from the currently played note,” we are basically attaching the “back end” of the span to a point of measurement in a motor performance, and finding out how far to the right the reader’s gaze extends at that point in time. This could be called the Forward Projective Approach of defining the span (see Fig. 2a below). Early pioneering studies using photographic methods applied this basic approach, both for the Eye-Voice Span in oral reading (35) and for the Eye-Hand Span in typewriting (36) and music reading (37). For each successive second in music reading, Weaver (37) measured how many notes or chords the eyes were ahead of the hands. Later, Sloboda (38, 39) used a variant of the Forward Projective Approach in an off-line setting, defining the Eye-Hand Span as the number of notes correctly played following a note on which the score was made invisible. With the advent of modern eye-tracking technology, the basic procedure has been to choose a point of measurement in the performance, find the fixation occurring concurrently with that point, and measure the distance between them in whatever units found suitable. An example would be Truitt and colleagues’ (32), p. 153, definition of the Eye-Hand Span as “the distance [in pixels] that the eyes were ahead of the executed note at the time the note was executed” (similarly in millimeters: (34); in number of notes: (29, 30, 31); in number of beats: (33)).

For addressing the Early Attraction Hypothesis, the Forward Projective Approach is inappropriate, since it is time-locked to action rather than perception (see Fig. 2a). That is, the measured spans are not defined for potential sites of visual interest lying ahead, but rather for motor actions corresponding to given notes of the score that are already being executed when the measurement is made. Hence the measured spans may, in fact, reflect the perception of other, upcoming notes, rather than perception of notes at the points of measurement. (The same is true in a variant substituting metrical beat onsets as points of measurement; see (4).) Another problem with the Forward Projective Approach is its imprecision: Whatever units of measurement the results are reported in, the initial pairing of the “hand” and the “eye” is here a pairing of two event onsets or dimensionless points in time that have not occurred exactly simultaneously. The fixation “in effect” during a key press might have had its onset some hundreds of milliseconds before the key press. Again, this just reflects that the measures discussed above are not meant to be exact about when, during the process, the first fixation to a given location appeared.

These problems can be addressed by what could be called the Single-Item Lag Approach to span measurement. Instead of pairing a point of time in the performance with a fixation that occurs approximately at the same time, here one pairs a fixation on a score element with the later performance of the very same element (this type of a definition for the Eye-Hand Span is given as the “formal” one by Holmqvist and colleagues [(40), p. 445–447]). That is, one basically chooses a note from the score and measures the temporal distance between a fixation to this note and the corresponding note onset in the performance (see Fig. 2b). Apart from providing measures that can be usefully defined for potential locations of visual interest, this strategy also gets rid of the problem of imprecision mentioned above. With such an approach, Furneaux and Land (29) reported that a “time index,” indicating the time interval between fixating a note and playing it, was reduced from ca. 1.3 s in a “slow” tempo to ca. 0.7 s in a “fast” one (while skill level, in particular, did not affect the time index). Rosemann and colleagues (33), in reporting similar tempo effects for the “eye-hand span in latency,” observed that while the measurement decreased for a faster tempo and increased for a slower one, the change was not quite proportional to the change in tempo. Unfortunately, the exact tempi were not reported in either of these studies, and the data sets only consisted of eight and nine pianists’ performances, respectively. Wurtz and colleagues (30) also applied the Single-Item Lag Approach in a study with seven violinists, but without controlling the tempo.

Among the studies using the Single-Item Lag Approach, only Rosemann and colleagues (33) made some effort to assess local, stimulus-driven changes in the Eye-Hand Span. Having intuitively rated each bar of their Bach keyboard score as “easy” or “difficult,” they used this sort of measurement and found no difference in the size of the spans measured for the two types of bars. Switching to a Forward Projective Approach, they did find that “difficult” bars received significantly smaller spans than “easy” ones. However, as explained above, the latter kind of results concern spans projected ahead from musical notes that are already being performed. Hence the results should not be taken to mean that more difficult items were initially glanced from a smaller distance than easier ones, but that performing difficult sections prevented the readers from looking as far ahead as they did while performing easier sections.

For the purpose of studying such stimulus effects as required by the Early Attraction Hypothesis, the Single-Item Lag Approach, too, has slight drawbacks. Most importantly, it is not only affected by the quickness of the reader’s eye-movement reactions to upcoming symbols, but also by her interpretive choices and possible failures in the performance domain. In recognizing a structurally weighty musical event in the score, the performer might come to emphasize it, say, by a slight deceleration before a metrically accented note (see, e.g., (41)). On the Single-Item Lag Approach, the resulting retarded note onset would yield a local increase in the Eye-Hand Span just because of the interpretive choice of the performer, even if the visual reaction to the note would not have been launched earlier on in the process than usual. Similar problems would be encountered if the performer commits errors in timing the note onsets. Overall, the measurement of the eye-movement response is here made contingent upon the success and accuracy of the motor response, disregarding the possibility that visual processing might also be successful when motor performance fails. A related problem afflicting any “hand”-based approach is that such approaches will not allow measuring spans for rests (symbols for silence), which might also be potential symbols of early interest (as they often indicate phrase boundaries). Finally, in the musical domain, some professionals might question the pedagogical applicability of measurements that do not allow conceptualizing “being ahead” in a snapshot-like manner, or in relation to the metrical time domain of the music, but instead require expressions like “you should glance at that note 1.5 s before you play it.”

For these reasons, we introduce a third approach to span calculation that can be called the Backward Projective Approach. In a nutshell, the idea is to start from a fixation and find the point at which the metrical time of music was running at fixation onset. The basic idea is illustrated in Fig. 2c. In working backwards from the landing sites of saccades, we measure the ability of individual symbols to catch the music reader’s eye, asking questions such as, “from how far back in the music will the musician first glance at this symbol?” Because we are not dealing with the “hand” of the performer, we prefer to call our measure the Eye-Time Span (henceforth, ETS). Note that with a mechanical performance, perfectly synchronized to the metronome (and with no rests as points of measurement), ETS would equal the Eye-Hand Span calculated on the Single-Item Lag Approach. However, in order to be sure that we are actually measuring visual reactions to the score, we prefer to use the ETS.

In research on oral reading, Laubrock and Kliegl (42) have used a similar spatial measure for the Eye-Voice Span, calculating the distance (in letters) of the currently articulated letter relative to each fixation onset. This is the only existing example of a Backward Projective Approach that we are aware of, but the difference is that Laubrock and Kliegl measure their span from fixations backward to spatial locations in the text defined on the basis of oral production. In the case of music with a metrical temporal framework, analogous spatial locations can be found irrespective of motor performance.

Calculating the ETS does not require synchronizing eye-tracking with a motor performance of the score, but only with one or more reference clicks (i.e. beat onsets) of the metronome governing the performance tempo. In comparison to “absolute” time, or clock time (measured in seconds), the metronome measures what can be called metrical time. With metrical time, we understand the succession of metrical beats that are typically organized in bars—both being temporal containers within which the notes can appear. To ensure temporal regularity in the performance, the passing of metrical time can be regulated with a metronome that is set to a particular tempo, say, 60 beats per minute (bpm). Hence a given stretch of metrical time, such as a 4/4 bar, can take different absolute durations: At 60 bpm, it would take 4 s, but at 100 bpm, its duration would be 2.4 s, etc.

Ideally, a notated score can be viewed as a visual graph of metrical time. If we read a simple score “as a metrical time scale,” imagining the metronome clicks to be horizontally “located” at the graphical quarter note symbols, any fixations landing on the score can similarly be assigned a position in metrical time. For instance, a fixation landing in the space between two note symbols, being horizontally one third of their mutual distance away from the symbol on the left, would be deemed 0.33 beats ahead of the beat onset on which the first note is supposed to be performed. This is an idealization of the relationship between metrical time and the score, but can be made to work in experimental settings, and leads to precise span measurements. In brief, then, the ETS for any fixation *F* is the distance, in beats, between the horizontal position of *F* in the musical score, and another—typically prior—position that corresponds to the point of metrical time at the onset of *F*. In our first simplified example of Early Attraction (Fig. 1), the four moments could now be seen as the onset times of fixations. If so, the metrical distance from each fixation back to the temporal cursor would correspond to the ETS for the fixations in question. For the first three fixations depicted (occurring at points of absolute time *t*_*1*_*–t*_*3*_), the ETS is 1.5 beats, while for the last fixation (at *t*_*4*_), it is 2.5 beats.

Proposing the ETS as a measure of visual and/or music-structural salience implies that we are primarily interested in measuring it for the first fixation falling on each notated symbol. In music reading, fixations might not land exactly on the note symbols, but near them (32, 43), and hence we need to determine an area of interest (AOI) around each symbol to find the first fixation on this area. In the following, AOIs will only be used to allocate fixations to note symbols. For any first fixation allocated to a symbol, the measurement of the ETS will be based on the actual horizontal location of the fixation.

### Aims

We are now in a position to operationalize the Early Attraction Hypothesis. We suppose that local increases in music-structural complexity (and thus visual salience) of the score may bring about local, stimulus-driven lengthening of the ETS. This type of effect has not been shown before, and hence it is not quite clear how this might happen, if it does at all. For orientation, we present two alternative sub-hypotheses that differ in terms of the accuracy of targeting the “looking-ahead-reactions.” According to the most straightforward, intuitive expectation, salient note symbols themselves catch the reader’s attention from a longer distance, provoking early oculomotor responses that result in relatively long ETSs for the elements in question. In this case, the ETS would turn out to function as a direct measure of music-structural (and/or visual) salience of the notated symbols to which the spans are anchored at the front end. Alternatively, it might be that spotting something challenging in the parafovea results in quickly fixating a bit closer toward the target. This might involve a saccadic range error in which saccades from more distant launch sites may “undershoot,” that is, fall short of their targets (17, 44). Note that if the perceptual span of a sight reader may extend 2–4 beats to the right from a given fixation (32, 34, 45), then a “looking-ahead-fixation” landing this much before the target element might, in fact, suffice for decoding the information at the target element, too. In any case, for early responses to salient targets, the alternative sub-hypothesis would suggest that the longer spans do not necessarily fall on the targets themselves, but rather on the areas preceding them. 

Notice that while both of these effects would support the Early Attraction Hypothesis, it would require a separate analysis of the length of incoming saccades to interpret them in terms of Distant Attraction. This is because, logically speaking, it would be possible for the reader to reach the difficult upcoming symbols with successions of shorter saccades, too. However, our working assumption is that any stimulus-driven effects of Early Attraction (shown by long local measurements of ETS) are most likely to come about by Distant Attraction (shown by measurements of long incoming saccades to the same areas). The phenomenon of Early Attraction cannot be measured by saccades only; nevertheless, understanding the specific eye-movement strategies in play requires saccadic analysis, in addition to the ETS.

In the following, we examine such potential music-structural effects on “looking ahead” in two sight-reading experiments. To give a balanced view of potential attraction effects, we incorporate two further variables that might conceivably affect the presence and extent of such effects. First, both of our experiments involve performances at two different controlled tempi. The effects of regulated tempo on looking ahead in music reading have been ill-studied (exceptions being (4, 29, 33)), but it is obvious that with a measure such as the ETS, tempo should be taken into account. This is because an increase in tempo shortens the absolute duration of the temporal buffer that a given ETS would allow the music reader for preparing motor performance. Thus we may expect ETS to increase with tempo to counterbalance this predicament. However, we can give no considered predictions on whether such tempo effects would interact with local stimulus complexity.

Second, considering that previous literature is not unequivocal about the influence of musical experience on the amount of looking ahead (e.g., (29) vs. (4)), our first experiment involves competent music readers with intermediate and high levels of expertise. Here, one might simply expect an overall effect of expertise in terms of longer ETSs for the more experienced musicians, but it is conceivable that such musicians would also be more sensitive to the local notated details, showing stronger effects of Early Attraction, as well. 

On purpose, we start with very simple sight-reading situations in which we expect musically competent participants to make few if any errors. We believe that if music reading is, to quote Sloboda’s (46), p. 235, memorable words, a “genuine species of music perception,” one should expect the visual processing of experienced readers to flexibly accommodate the features of the notated stimuli—also in circumstances in which reading is effortless and the readers need not function at the limits of their capacities.

## Experiment 1

### Method

*Participants.*Our original number of participants (40) was cut down to 37 by missing eye-movement data in two cases, and by highly exceptional ETS measurements in one case. The 37 participants included (a) 14 students (9 females, 5 males) of music performance at a Finnish conservatory and (b) 23 musically active education majors (15 females, 8 males) minoring in music education at the department of teacher education of a Finnish university (incl. one health care major with a degree in cello performance). The two groups are henceforth titled “performance majors” and “education majors,” respectively. The participants were between 17 and 36 years old, the average ages being 24.4 years for the performance and 25.8 years for the education majors (*SD* = 4.6 years for both groups).

Although admission to both study programs required passing program-specific tests of musicality and musical performance, the two participant groups were considered to represent different levels of musical expertise: The performance majors were under full-time training to become professional musicians and/or instrumental teachers, whereas for the education majors, instrumental performance was only one part of their minor subject studies (the study curriculum aims to train the students to give classroom music lessons) and a hobby. All but one participant (an education major) included the piano in their personal list of instruments. Out of 14 performance majors, 13 had completed professional-level piano degrees, and one an elementary-level degree; 11 of them reported the piano as their main instrument. Likewise, 11 of the education majors marked the piano as their main instrument, and 11 had completed piano degrees on the professional (6) or elementary (5) level. The performance majors, on average, reported slightly more years of active piano playing (*M* = 14.8, *SD *= 5.2) than the education majors (*M* = 11.3, *SD *= 6.6), but the difference was not significant according to an independent samples *t*-test (*t*[32] = –1.649; *p *= .109). Participation was voluntary and rewarded with a cafeteria voucher or course credit.

**Fig 3.**
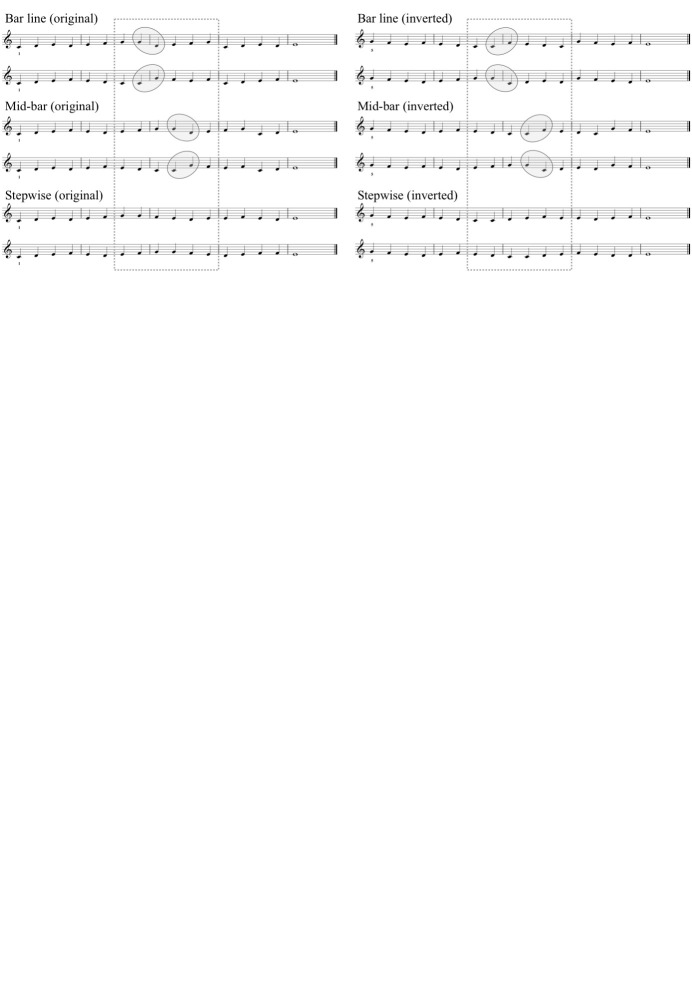
The 12 stimulus melodies in Experiment 1. The grey bubbles are here added to indicate the intervallic skips involved; The dashed line circumscribes the six notes that were taken into account in the analysis.

*Stimulus Materials.*The stimulus set consisted of 12 five-bar melodies notated in G-clef in 4/4 time, each of them using the first five diatonic pitches of the C major scale (see Fig. 3). Three separate sets of four melodies were included, one for each of the conditions: Bar line, Mid-bar, and Stepwise (the total set of melodies differed from the one in Penttinen & Huovinen [2, 15] by the addition of the Stepwise condition). Each condition involved four stimuli: two original melodies beginning on C4 and two corresponding diatonic inversions beginning on G4 (i.e., “upside-down versions” of the same melodic contour). All of the melodies ended on an E4 whole note after four bars of continuous quarter notes. The fingering for the first note was indicated by numbers “1” (index finger on C4) or “5” (little finger on G4) in order to ensure that the participants would not need to move their hand during playing. The Stepwise melodies consisted of entirely stepwise successions of notes, while the Bar line and Mid-bar melodies had two larger intervals of a perfect fourth and a perfect fifth placed at either the bar lines of bar 3, or two quarter-note beats after them. The Stepwise melodies closely followed the melodies in the two other conditions, including the note repetition that was needed in the Bar line and Mid-bar conditions to place the skips at the intended locations, but also included another note repetition required for ending on E4.

The stimulus melodies were written with Sibelius music notation software, setting the note stems exactly at equal 15 mm-distance (0.59 in) from one another, and the bar lines exactly at the midpoint between two note stems. The height of the staff system was 9 mm (0.35 in) and the width of bars 2, 3 and 4 was 60 mm (2.36 in).

For presentation in the experiment, the melodies were organized into four different presentation orders of 12 trials each. Each presentation order was subject to the requirements that (i) no two successive melodies would represent the same condition (Bar line, Mid-bar, Stepwise), and (ii) within the succession of 12 melodies, both consecutive sets of six melodies would always include one of the original melodies for each condition, as well as its inversion. 

*Apparatus.* Eye-movement recordings were conducted using a Tobii TX300 Eye Tracker (Tobii Technology AB, Stockholm, Sweden). Both eyes were tracked with a sampling rate of 300 Hz, and with an accuracy of 0.4 degrees (binocular). For presenting the stimuli, we used a 23” widescreen TFT monitor with a screen resolution of 1,920 x 1,080 pixels. The participants were seated with their eyes approximately at a 65 cm distance from the screen. Their performances on a Yamaha electric piano were recorded using the Power Tracks Pro Audio sequencer software that also provided the metronome click.

*Procedure*. The participants were randomly assigned to the four presentation orders of the stimuli by letting them select suitable times for the experimental session themselves, and by rotating the presentation orders between successive participants. The experiment was conducted individually for each participant, in the presence of one experimenter (the third author).

**Fig 4.**
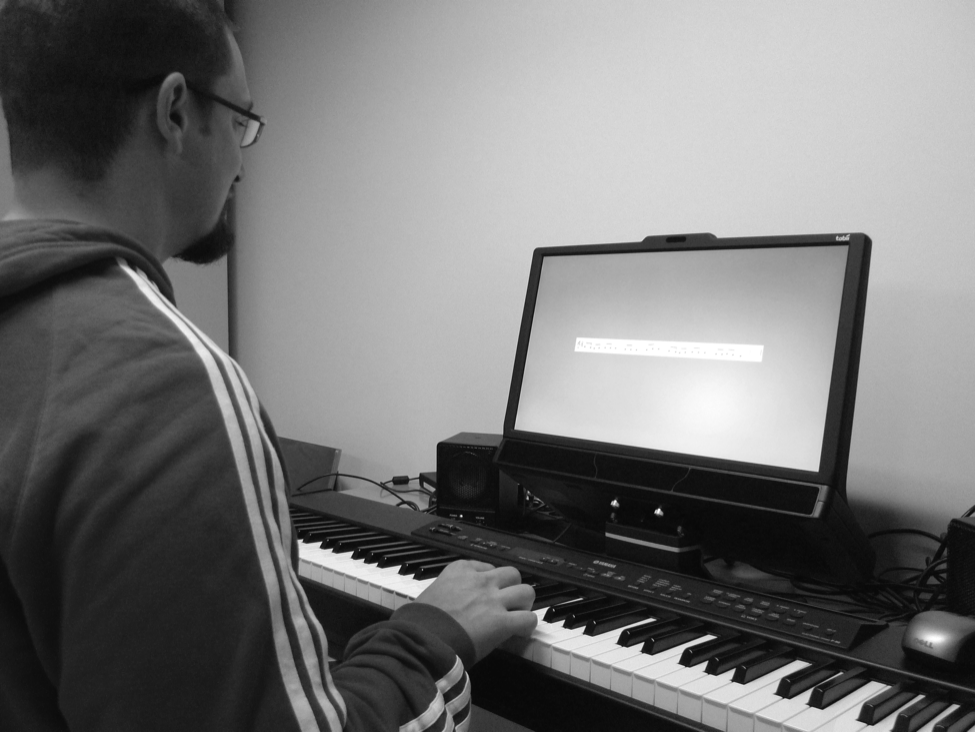
The setup of the eye-tracker and the electric piano in Experiment 1, demonstrated by a colleague of the authors. A similar setup was applied in Experiment 2.

On entering the laboratory, each participant was first asked to fill out a written questionnaire about his/her musical background, and was then introduced to the laboratory setting in which a computer screen was positioned right behind a keyboard, assuming the role of a music stand (Fig. 4). After allowing the participant to adjust the piano seat at a comfortable height, a five-point calibration procedure was carried out, and the participant was asked to perform two practice trials incorporating melodies similar to the ones used in the experiment, using the right hand only, at the tempo given by the metronome set at 60 bpm. The practice trials acquainted the participant with the research protocol in which the metronome would be constantly ticking, written instructions about the procedure would appear on the screen between the melodies when needed, and the location of the first melody note for each trial would always be indicated in advance by an “X” appearing on the screen two metronome clicks before the staff appeared. The participant was instructed to wait for two more metronome clicks after the appearance of the staff before starting the performance. 

After a new calibration, the 12 experimental trials followed the procedure of the practice trials, except that the first six melodies would always be performed at the tempo of 60 bpm, and the last six at 100 bpm. The participant only played each melody once. The experimenter switched the images (including the notated stimulus melodies) on the screen by pressing the space bar on a separate computer, synchronizing her actions with the metronome clicks.

### Data analysis

*Data set.* Our aim was to analyze what happened in error-free “model performances” of the stimuli in and around the area that (in two of the conditions) included the larger intervallic skips. First, then, we restricted our data set to correct performances by excluding all trials that included any clear performance errors, defined as wrong notes appearing instead of, or in addition to, the notes specified by the notated score of the given trial. By using MIDI information to analyze the 444 trials, we identified 17 trials with one or more such errors, leaving us with 427 successful performances. Second, in order to minimize, as far as possible, any effects of beginning or ending the melody (2), we further restricted all of our analyses to the six quarter notes (i.e., six AOIs) appearing within the area marked with a dashed line in Fig. 3.

A fixation was defined according to the default setting of Tobii Studio 2.2.8, with velocity and distance thresholds of 35 pixels/sample. Only fixations targeting the staff system and related to the actual reading of musical notation were included in the analysis, and so the AOIs only extended vertically to a 35-pixel distance (9.5 mm/0.38 in) from the outermost staff lines. The limit was set in an explorative manner, with the goal of excluding clear outliers while including as many potentially task-relevant fixations as possible. With such a limit, 89.1% of all fixations between the first and last note onsets in the trials fell within this visual area. For assigning fixations to particular note symbols, the visual field corresponding to the second half of bar 2 and the entire bar 3 was then segmented into six, rectangular areas of interest (AOIs), equal in size, and each corresponding to a quarter-note symbol. The lines between AOIs were drawn exactly between the note stems. 

Based on the first fixations targeting the AOIs, measurements of ETS and incoming saccade length were assigned to the six notes in the analyzed bars. For better comparability, both ETS and incoming saccade length were analyzed for the same set of first fixations to AOIs. For this purpose, we left out any first fixations that corresponded to (i) negative measurements of ETS (six; 0.25 %) and (ii) regressive incoming saccades (144; 6.01 %), both of which would be irrelevant for our theoretical concerns. Furthermore, we also left out (iii) first fixations for which the incoming saccade would be longer than the corresponding ETS measurement plus two beats (19; 0.79 %). This was to practically discard long saccades arising in situations in which the reader would have glanced back from the currently played notes (say, to check the key signature in the beginning of the line), followed by a long incoming saccade back to the point of reading. The excluded fixations, as well as the few above-mentioned trials with performance errors, were regarded as data missing completely at random. A total of 2,232 first fixations were left for the analysis of ETS and incoming saccade length. Notice that this is the subset of fixations for which our measurements were to be defined, but, for measuring incoming saccades, the full original set of fixations was left available to provide information concerning prior saccade launch sites. Saccade lengths were thus calculated as horizontal distances to the previous fixation. To ensure comparability between our two measurements, we converted saccade lengths from pixels to metrical units (beats).

Our measurements required synchronizing each participant’s eye-tracking data with the metronome clicks that had guided the performance (while the performance data from the piano could be ignored). The eye-tracking recordings included timestamps for the computer key presses with which the experimenter had switched the screen images on the metronome clicks. (In this respect, the experimenter showed relatively good accuracy: The experimental design involved six pairs of timestamps ideally produced 2 s apart; The 95% confidence interval for durations between them was [2001.6 ms, 2012.1 ms].) For each participant, we synchronized the eye-movement data with the metronome by taking all of these 12 timestamps in the tempo of 60 bpm, and by finding the median of the decimal parts of a second in these timestamps. This yielded an approximated reference value for the cyclically recurring beat onset, i.e., the metronome click. Using the reference value for the beat onset, all fixation onsets could be indexed with their metrical time of appearance in terms of metrical beats (of the score) and decimal parts thereof. For instance, if the metronome click is approximated to appear at 300 ms after each full second in the recording, if the performance begins at 12.3 s into the recording, and fixation *F* occurs, say, at 14.5 s in the recording (i.e., 200 ms after the second beat onset), then the metrical time of appearance for *F* is given as 2.2 beats. Since the tempo change to 100 bpm had been pre-programmed in the sequencer software, the reference value for the beat onset in the second tempo could be similarly determined from the above-mentioned timestamps.

*Statistical analysis.* The data were analyzed by using generalized estimating equations (GEE)—an approach extending generalized linear models (GLMs) for longitudinal and correlated data (47, 48). This approach leads to population average models (marginal models) where the interest lies in regression parameters instead of variation parameters. The method was applied because the data were correlated within individuals, due to the study design, and because the distributions of the ETS and incoming saccade lengths were skewed. The analyses were carried out using *R* software (49) with the package “geepack” (50, 51, 52). We assumed a common correlation within observations, i.e., that each pair of a given participant’s observed values has approximately the same correlation. Note that we retained the individual observations instead of taking an average within a participant: Optimally, we thus had 72 (6x12) observations from each participant. The independent variables considered in the analysis were Tempo (60 bpm, 100 bpm), Expertise (performance majors, education majors), Condition (Stepwise, Bar line, Mid-bar), and Note (1–4). After these, the analysis involved the interaction Condition:Note (representing the precise effects of the melodic skip), all two-way interactions involving Expertise, as well as the three-way interaction Expertise:Condition:Note. For each analysis, the non-significant interaction terms involving Expertise were discarded from the final model. For all variables, we focus on interpreting the highest-order interaction of the final model, if significant. The estimated parameters of the fitted models are given in Appendices 1–2. (Note that the coefficients in the Appendix are always reported with respect to a reference level as in regression models. The comparisons made in the following Results section cannot thus be directly read off from the Appendices.) Pairwise post hoc comparisons of predictions resulting from GEE were conducted using the package “emmeans” (53) in *R*, adjusting for multiple comparisons.

### Results

*Eye-Time Span*. The mean ETS observed in the experiment was 2.12 beats (SD = 0.90, Mdn = 1.97). Given the skewness of the distribution (moment coefficient of skewness 2.28), we assumed a gamma distribution, and carried out a GEE analysis of the ETS by applying the inverse link function and exchangeable correlation structure. The Wald statistics on the fitted model are shown in Table 1. There were significant main effects of Expertise and Tempo, a nearly significant main effect of Condition, and, most importantly, a significant interaction between Condition and Note. Regarding the main effect of Expertise, the model indicates that the performance majors, on average, operated with longer ETS than the education majors. According to the predictions of the model, the difference in ETS between the two groups varied from 0.29 to 0.53 beats. The main effect of Tempo, in turn, indicated 0.21–0.41 beat longer spans at 100 bpm than at 60 bpm. This simply reflects the fact that when tempo increases (here, with a factor of 1.67), the reader will still need to allocate some reasonable time resources for symbol decoding and motor planning: Consequently, the ETS may also tend to increase at least somewhat (here, with a factor of around 1.1–1.2).

**Table 1.** Wald statistics for the GEE analyses of first fixations to AOIs in Experiment 1.

	Eye-Time Span				Incoming Saccade Length		
	*df*	Χ²	*p*		*df*	Χ²	*p*
Expertise	1	11.66	< .001***		1	7.66	.006**
Tempo	1	22.93	< .001***		1	0.00	.978
Condition	2	5.94	.051		2	0.76	.683
Note	5	6.65	.248		5	63.70	< .001***
Condition:Note	10	67.53	< .001***		10	26.73	.003**

*** *p*< .001, ** *p* < .01, * *p* <.05

**Fig 5.**
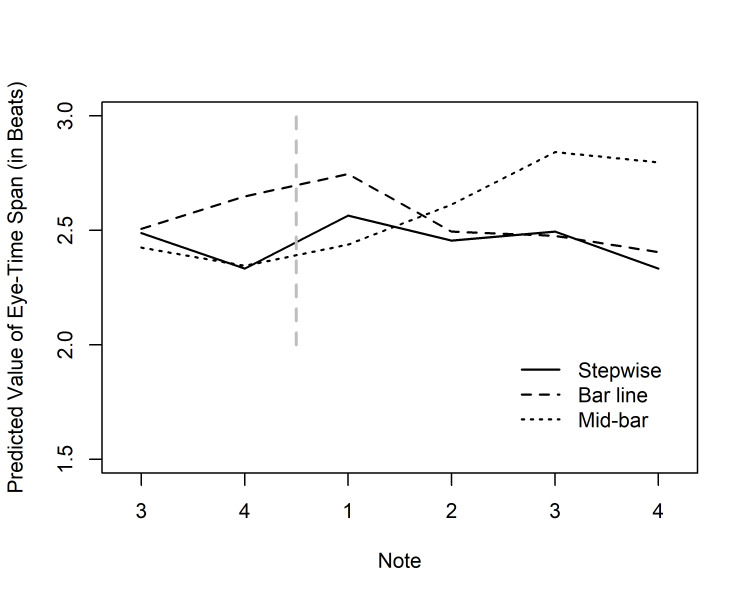
Predicted values of ETS (by Condition and Note) for the group of performance majors in the tempo of 100 bpm in Experiment 1. ETS values are given for notes 3–4 of bar 2, and for notes 1–4 of bar 3 (cf. Fig. 3); The vertical dashed line represents the bar line cutting across this area.

The interaction between Condition and Note indicates local, stimulus-driven effects on the length of the ETS. Predicted values for performance majors in the tempo of 100 bpm are plotted in Fig. 5. In the Mid-bar condition, the highest peaks appeared on the notes following the skip. For these two notes, Tukey’s post hoc tests showed the value for the Mid-bar condition to differ significantly both from the Stepwise (bar 3, note 3: *p* = .048; note 4: *p* = .022) and the Bar line values (bar 3, note 3: *p* = .011; note 4: *p* = .012). In the Bar line condition, the clearest peak likewise appeared directly following the skip, on the first note of bar 3; Tukey’s tests indicated a significant difference from the Mid-bar value (*p* = .016), although not from the Stepwise condition that seemed to show some meter effect on the downbeat. However, in the Bar line condition there was a high value also on the note preceding the skip (bar 2, note 4), with significant differences to the Stepwise (*p* = .001) and Mid-bar conditions (*p* < .001). All other paired comparisons were non-significant (*p* > .05). In sum, ETS peaked exactly at the notes that we had assumed to exhibit points of local music-structural complexity, as well as points of heightened visual salience, but high values were also observed at the preceding note in the Bar line condition. All of these results are in line with the Early Attraction Hypothesis.

**Fig 6.**
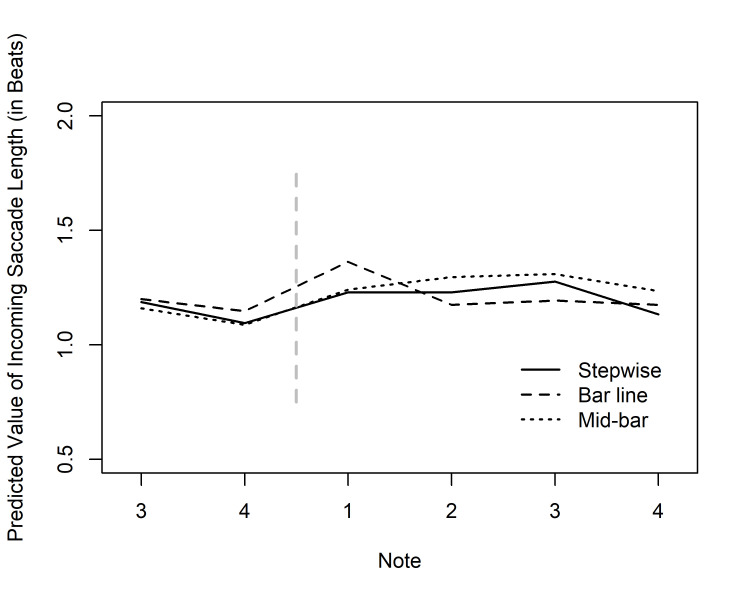
Predicted values of incoming saccade lengths (by Condition and Note) for the group of performance majors in the tempo of 100 bpm in Experiment 1. The values are given for notes 3–4 of bar 2, and for notes 1–4 of bar 3; The vertical dashed line represents the bar line.

*Incoming saccade length*. The incoming saccades to AOIs had an average length of 1.13 beats (SD = 0.55, Mdn = 1.02). In order to test if the melodic skips also affected the length of incoming saccades, we carried out a separate GEE analysis for the incoming saccade lengths at first fixations. Assuming a gamma distribution due to skewness (moment coefficient of skewness 5.46), we applied the inverse link function and exchangeable correlation structure. The results showed significant main effects of Expertise and Note, as well as a significant interaction between Condition and Note (see Table 1). The expertise effect indicated longer incoming saccades for the performance majors. According to the predictions from the model, the difference in incoming saccade length between groups varied from 0.09 to 0.14 beats. The predicted values are shown in Fig. 6, and again, they appear to reflect the melodic skips. According to a Tukey’s test, the note after the skip in the Bar line condition (bar 3, note 1) received significantly longer incoming saccades than the same note in the Stepwise condition (*p* = .031). Likewise, the note after the skip in the Mid-bar condition differed significantly from the corresponding note in the Bar line condition (*p* = .011). All other differences were non-significant. These results, although somewhat milder than those for the ETS, are nevertheless clearly in line with the Distant Attraction hypothesis that expects the visually and music-structurally most salient notes to attract saccades from relatively distant launch sites.

**Fig 7.**
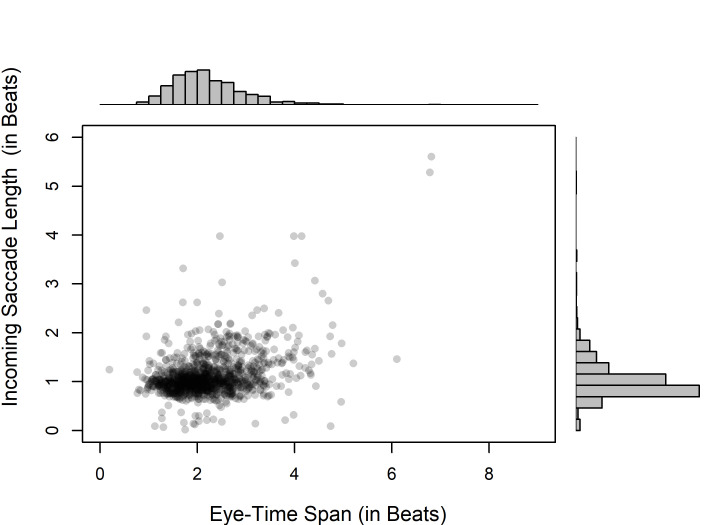
The relationship between incoming saccade length and ETS (with histograms for both variables) at the tempo of 100 bpm in Experiment 1.

Pooling all participants and conditions together, we may look at the relationship between ETS and the length of incoming saccades. According to Spearman rank correlations, there was a significant positive association between these measurements both in the tempo of 60 bpm (*ρ*[1,117]= 0.317, *p* < .001), and in the tempo of 100 bpm (*ρ*[1,111]= 0.282, *p*< .001). Although this analysis does not take into account within-subject correlation, it seems that a long ETS is quite often due to an extended incoming saccade. As exemplified in Fig. 7 for the higher of the two tempi, typical ETS measurements of around 2 beats might often correspond to incoming saccade measurements of around 1 beat, but longer ETS measurements would often correspond to slightly longer incoming saccades.

### Discussion of Experiment 1

This study addressed local, stimulus-driven effects on “looking ahead” in simple sight-reading tasks. The results indicate that ETS measurements may be sensitive even to small changes in the relative complexity of musical material. By interspersing stepwise, diatonic melodies with melodic skips of a fourth or a fifth, we could elicit significant variations in ETS between quarter notes, as suggested by the Early Attraction Hypothesis. The predicted values given by GEE suggested that first fixations to notes were typically most ahead of the metrical time just after the skip (see Fig. 5). This shows a direct Early Attraction effect to the notes that were expected to represent the highest music-structural complexity, as well as visual salience. In one of the skip conditions, high values were also observed just before the skip, which gives qualified support for the idea that Early Attraction could be seen even on notes preceding the local complexity.

In order to interpret such Early Attraction, we also looked at incoming saccade lengths at the same AOIs (i.e., for the same set of first fixations on note symbols). Again, the placement of the melodic skips had a significant effect on the measurements: In both skip conditions, long incoming saccades, differing significantly from at least one of the other conditions, were observed at the notes following the skip. This is in line with the Distant Attraction Hypothesis, although we may note that the differences between the conditions were smaller than for the ETS results. This is not surprising, as the readers’ gaze tended to proceed from note to note, and this measurement took into account the distance between two fixations. Over and above the local effects of the melodic conditions, we also observed highly significant overall correlations between incoming saccade lengths and ETS measurements. All of this suggests that, even in trivially simple sight-reading tasks such as these, the local variability of ETS is at least in part due to long forward saccades, and that a notable part of such saccades represents quick reactions to upcoming, visually salient complexities in the musical structure.

In addition, our results indicate tempo- and expertise-related increases in ETS, but neither of these variables interacted with the local stimulus-driven effects. While the overall tempo effect was in the expected direction, and requires no further comment, the effect of expertise is more notable. In both of the two performance tempi, professional musicians (performance majors) generally used larger ETSs than amateur musicians (education majors). In the literature on the Eye-Hand Span, it has previously been suggested that such a measure—when determined using the Single-Item Lag Approach—would be largely independent of the skill level of the performer, with average values somewhere around one second (29, 30). In a study with controlled performance tempi, a Forward Projective Approach to span measurement, and span lengths roughly categorized on the level of metrical beats, Penttinen et al. (4) nevertheless found experienced musicians to use longer spans more often than was the case for amateur musicians. The present results—obtained using a more accurate Backward Projective Approach—similarly suggest that expert music readers may, indeed, look farther ahead in the music than less proficient individuals. Given the possibility to read our stimulus score “as a time scale,” and given the controlled tempi, our results suggest an absolute time difference of 300–400 ms in the medians between the two participant groups, depending on tempo (with medians of 2074 ms and 1492 ms for the more experienced group, in the tempi of 60 bpm and 100 bpm, respectively). These findings differ from the earlier Eye-Hand Span measurements, and for a good reason: In determining the ETS for first fixations to a given area, we are focusing on the subset of spans in which any stimulus effects should be reflected, and in which any skill differences are consequently most likely to surface. Nevertheless, the lack of any significant interactions involving Expertise, Condition, and Note suggests that the observed stimulus-driven effects were already in place with our intermediate group of education majors.

 In sum, then, the results of Experiment 1 would seem to lend support to the notion of Early Attraction (“looking ahead” to points of local complexity), as well as to that of Distant Attraction (long saccades to points of local complexity). The results suggest that even small local irregularities in the notated stimulus may lead competent music readers to anticipate the potential processing difficulty by quickly looking farther ahead. This leaves open how the situation would change if the local complexities would be increased: Would the effect remain the same, or would it be stronger? Experiment 2 was hence designed with the purpose of teasing out stronger stimulus-driven effects by increasing the visual saliency and music-structural complexity of the target. At the same time, we wanted to embed the targets in longer sight-reading tasks, giving the reader more time to establish a suitable processing style. It was supposed that an intensification of the “visual irregularity” at the target element might bring about an even clearer result in terms of long forward saccades to (or toward) the target note, also resulting in larger ETSs at the target. As our main interest lies in longer, stimulus-driven spans, and given that Experiment 1 suggested such looking-ahead reactions to be pronounced in expert musicians’ reading, we chose to focus exclusively on highly experienced music readers.

## Experiment 2

### Method

**Fig 8.**
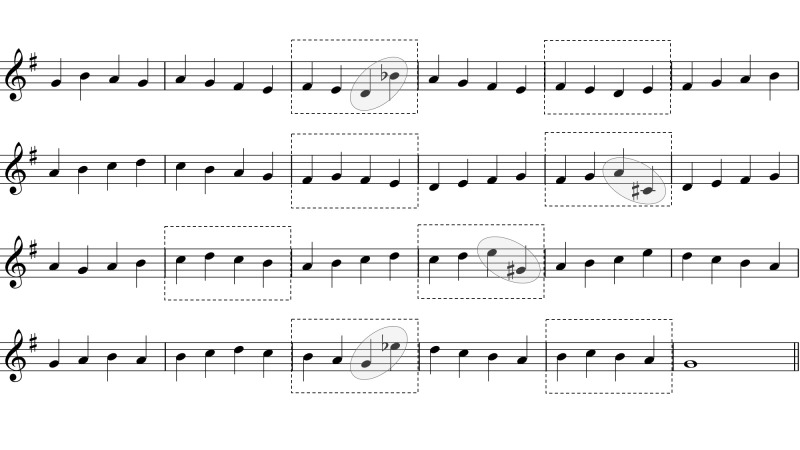
One of the eight stimulus melodies applied in Experiment 2. The dashed rectangles are here added to mark the Skip and Stepwise bars used in the data analysis; the grey bubbles are added to indicate the intervallic skips involved.

*Participants.* The original group of participants consisted of 26 professional piano students from three Finnish universities. For some individuals, performing the 32-bar melodies of Exp. 2 (see Fig 8) resulted in head movements during performance and therefore calibration difficulties. We thus conducted a detailed video-based pre-analysis of the eye-movement recordings, in which the data set was checked for its quality row by row (total of 32 staves of music notation for each of the 26 participants; see Stimulus materials, below). In order to get enough points of measurement from each participant for a comprehensive data set, we decided to include the 14 participants for whom the pre-analysis indicated no precision errors. The following analysis is thus based on 14 participants (incl. 12 females, 2 males) whose data did not contain missing events or poor data quality.

11 of the 14 participants already had a prior conservatory or university degree in piano performance. Their ages varied between 20 and 58, with an average of 28.2 years (*SD* = 9.5). The reported years of playing the piano varied between 7–42, with a group average of 19.1 years (*SD* = 8.7). The participants reported an average of 14.1 hours of weekly music-making (*SD* = 9.8) and 15.4 hours of weekly music reading (*SD* = 10.9), the latter figure including playing from written notation, silent music reading, and any other notation-related activities such as writing scores or teaching notation. Participation was voluntary and rewarded with a cafeteria voucher.

*Stimulus Materials.* The stimulus set for Experiment 2 comprised eight mostly stepwise melodies in 4/4 time, each consisting of merely quarter notes, laid out in four staves, six bars per staff (see Fig. 8). The melodies were divided into two sets of four so that there would be two sets of different original melodies in the keys of G, C, F, and Bb. On each of the four staves in each melody, one larger intervallic skip (minor sixth) was inserted in one of the bars 3–5. The latter note in each skip was chromatically altered with an accidental, and this target note was always the last note in its bar. In each melody, the four targets constituted upper and lower chromatic neighbors leading to scale degrees II and V of the key. Each skip was preceded by at least one bar of stepwise movement, reversing at the skip. After the skip, the registral direction of the melody was again reversed by a stepwise melodic progression “filling the gap” (as is conventional in tonal musical styles; see 54). For half of the melodies, the first staff system had the target note in the third bar, and for half of them, it was in the fourth bar. In each melody, the same target position was also used for the last staff system, and the two middle systems had the target in bars 4 and 5, or in bars 3 and 5, complementing the first and last targets in bars 3 or 4. As seen from the example of Fig. 8, the rest of each melody would be filled in by stepwise movement, beginning and ending on a tonic note, avoiding repeating notes, two-note patterns and consecutively repeated bars (and inserting one interval of third in each system, if required to reach the next target pattern or final note with a stepwise movement). Note that now there was no simple correspondence between the five fingers and specific notes, as in Experiment 1, but that the participants had to find their fingerings on the fly, making for somewhat more challenging, but realistic performances.

For presentation in two experimental conditions, one of the sequences of melodies in the keys of G, C, F, and Bb was assigned to the tempo of 60 bpm, while the other sequence was assigned to the tempo of 100 bpm. To counterbalance effects of the specific melodies and participant fatigue, four stimulus sets were assigned to the two tempo conditions in a 2x2 design by switching between the two sequences of melodies, as well as the internal order of the melodies in a sequence (and thus the keys: G–C–F–Bb or Bb–F–C–G).

The stimulus melodies were written with Sibelius music notation software. The height of the whole four-staff system, when presented on the screen, was 127 mm (5.0 in; 10.5 mm [0.4 in] for one staff system), and the width was 299 mm (11.8 in). The distance between bar lines was 48 mm (1.9 in). Within the analyzed bars (see Fig. 8), the centers of two note heads were 11 mm (0.4 in) apart. Across a bar line, due to a more natural layout, this distance was 15 mm (0.6 in).

*Apparatus.* Eye-movement recordings were conducted using a Tobii T60XL Eye Tracker (Tobii Technology AB, Stockholm, Sweden). Both eyes were tracked with a sampling rate of 60 Hz, with an accuracy of 0.5 degrees. For presenting the stimuli, we used a 23” widescreen TFT monitor with a screen resolution of 1,920 x 1,200 pixels. The participants were seated with their eyes approximately at a 65 cm distance from the screen. Their performances on a Yamaha electric piano were recorded using the Logic Pro X sequencer software that also provided the metronome click.

*Procedure.* The participants were randomly assigned to the four stimulus sets by letting them select suitable times for the experimental session themselves, and by rotating the presentation orders between successive participants. The experiment was conducted individually for each participant, in the presence of one experimenter (the third author). The procedure in the laboratory was similar to that applied in Experiment 1. During the task, the participant constantly heard a metronome click. After a slide naming the key of the upcoming melody, an “X” would always appear on the screen four beats prior to the appearance of the staff; the participant was instructed to start playing after two more beats. Two melodic stimuli, following similar compositional rules as the experimental items, but in the keys of D and Eb, were used as practice items in the tempo of 60 bpm. After this, the participant performed the first four melodies in the tempo of 60 bpm, after which there was a tempo change in the metronome, and four other melodies were performed at 100 bpm.

### Data Analysis

*Data set.* The eye-tracking was subject to some data loss in that on the lowest staff in the stimuli, some of the fixations fell below the stimulus image and were left unrecorded. To be conservative, the following analysis is based on the first three staves. Each of the target bars involving a larger intervallic skip was counted as one experimental item for a Skip condition, and hence every participant contributed eye-movement data concerning 12 such items at 60 bpm and 12 items at 100 bpm. In addition, a Stepwise condition was created by sampling an equal number of bars with only stepwise melodic movement (for each staff system with the target being at bars 3, 4, or 5, the Stepwise bar was picked at bars 5, 2, or 3, respectively; see Fig. 8). As in the first experiment, the data set was restricted to correct performances by excluding bars with any performance errors (defined as before). Excluding eight Skip or Stepwise bars with one or more errors, and one whole trial because of a tempo error, the data set consisted in the eye-movement recordings for 330 correctly executed Skip bars and 328 correctly executed Stepwise bars.

A fixation was defined according to the default setting of Tobii Studio 3.3.0, with velocity and distance thresholds of 35 pixels/sample. In the score, the Skip and Stepwise bars were segmented into four rectangular AOIs each, drawing the lines between the AOIs at bar lines and, within each bar, at the exact midpoints between the note heads (stems were not used for this purpose now, as they would flip on the left side of the note head in a higher register). Due to the more realistic score layout compared to Experiment 1, the first and last AOIs of each bar were somewhat narrower and broader than the others, respectively, but this was deemed immaterial given that the same layout was used in both conditions.

As in Exp. 1, measures of ETS and incoming saccade length were determined for the same set of first fixations to AOIs. As before, we left out all such fixations for which (i) ETS was negative (seven; 0.31 %), (ii) the incoming saccade was regressive (182; 8.11 %), or (iii) the incoming saccade was longer than the corresponding ETS measurements plus two beats (42; 1.87 %). A total of 2,014 first fixations were left for the analysis of ETS and incoming saccade length. The excluded fixations, as well as the few above-mentioned bars with performance errors, were regarded as data missing completely at random.

The eye-movement data were synchronized with the metronome using 12 timestamps from the experimenter’s key presses as in Exp. 1. (For each participant, these included 4 pairs of timestamps ideally produced 4 s apart when the “X” was visible on the screen; The 95% confidence interval for these time intervals was [3974.6 ms, 4004.3 ms], suggesting relatively good accuracy.)

**Table 2.** Wald statistics for the GEE analyses of first fixations to AOIs in Experiment 2.

	Eye-Time Span				Incoming Saccade Length		
	*df*	Χ²	*p*		*df*	Χ²	*p*
Tempo	1	1.06	0.303		1	1.48	.223
Condition	1	29.40	< .001***		1	11.85	< .001***
Note	3	11.07	.011*		3	12.72	.005**
Condition:Note	3	13.71	.003**		3	6.71	.082

*** *p*< .001, ** *p* < .01, * *p* <.05

**Fig 9.**
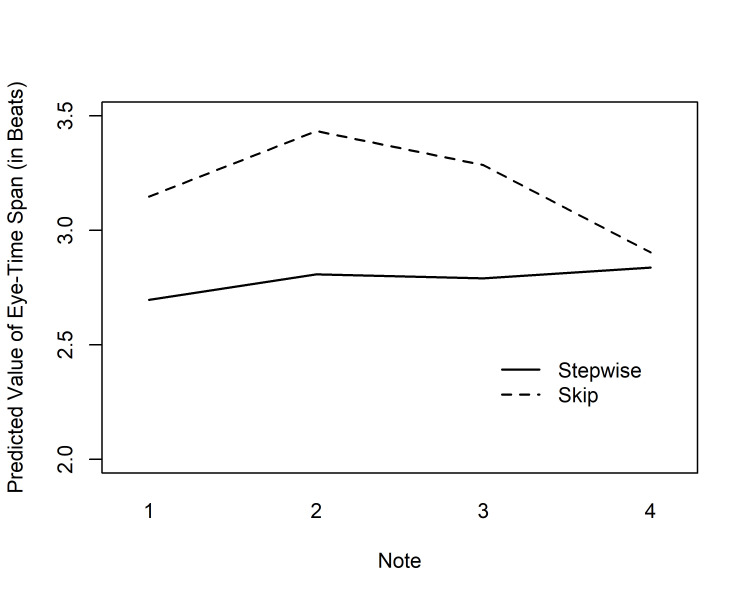
Predicted values of ETS (by Condition and Note) in the tempo of 100 bpm in Experiment 2. In the Skip condition, Note 4 was the target following the skip

*Statistical analysis.* The data were analyzed following the same procedure as in Exp. 1, except that now the variables only included Tempo (60 bpm, 100 bm), Condition (Stepwise, Skip), and Note (1–4), as well as the interaction Condition:Note. The estimated parameters of the fitted models are given in Appendices 3–4. As above, we will focus on interpreting the highest-order interaction, when significant.

### Results

*Eye-Time Span*. The average ETS was 2.93 beats (SD = 1.32, Mdn = 2.70). Fitting a GEE model proceeded according to the same technical details as outlined for the first experiment, using a gamma distribution due to the skewness of the distribution (moment coefficient of skewness 2.28). The results are shown in Table 2. The main effect of Tempo was not significant. Instead, there were significant main effects of Condition and Note, and, most importantly, an interaction between Condition and Note. Tukey’s tests showed significant differences between the two conditions on notes 1–3 (all *p*s < .001), but not on note 4 (*p* > .1). Predicted values for the four notes are shown in Fig. 9. What is seen here is that the impending, relatively more salient element on the fourth beat of the bar has generally led the participants, on a group level, to use longer spans on the first three beats of the bar. Notably, then, the significant Condition:Note interaction was not generally due to longer spans being targeted to the salient point of structural complexity at the fourth note. Rather, the notes preceding this point received longer spans. These results are in line with the Early Attraction Hypothesis, but suggest that the ETS effects of upcoming difficulties may not only be recorded on the relatively complex symbols themselves, but also on the previous ones.

**Fig 10.**
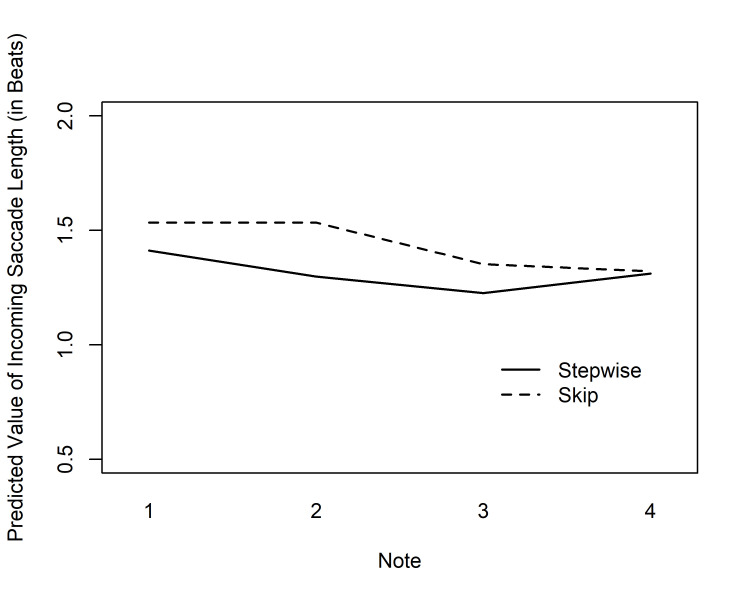
Predicted values of incoming saccade length (by Condition and Note) in the tempo of 100 bpm in Experiment 2. In the Skip condition, Note 4 was the target following the skip.

*Incoming saccade length*. The average length of incoming saccades was 1.39 beats (SD = 0.63, Mdn = 1.28). In order to analyze the effects of the melodic condition on incoming saccade length, we also carried out a GEE analysis for this dependent measure (the technical details being as in Experiment 1; moment coefficient of skewness 2.38). The results are given in Table 2. Like in the analysis of ETS, there was no significant effect of Tempo, but main effects of Condition and Note were observed. Unlike in the ETS analysis above, however, the interaction between Condition and Note remained non-significant. In other words, while there was a clear indication of the Skip condition bringing about longer saccades (Mdn = 1.33 beats) than the Stepwise condition (Mdn = 1.21 beats), these saccades did not uniformly land on a given note in the bar. Nevertheless, the pattern of predicted values (Fig. 10) is reminiscent of that for the ETS (Fig. 9) in that group-level differences between the two conditions are already seen on notes preceding the final, “most complex” note. The saccadic results thus support the Distant Attraction Hypothesis, but only as a general complexity effect that might not be found exactly on the complex elements themselves.

**Fig 11.**
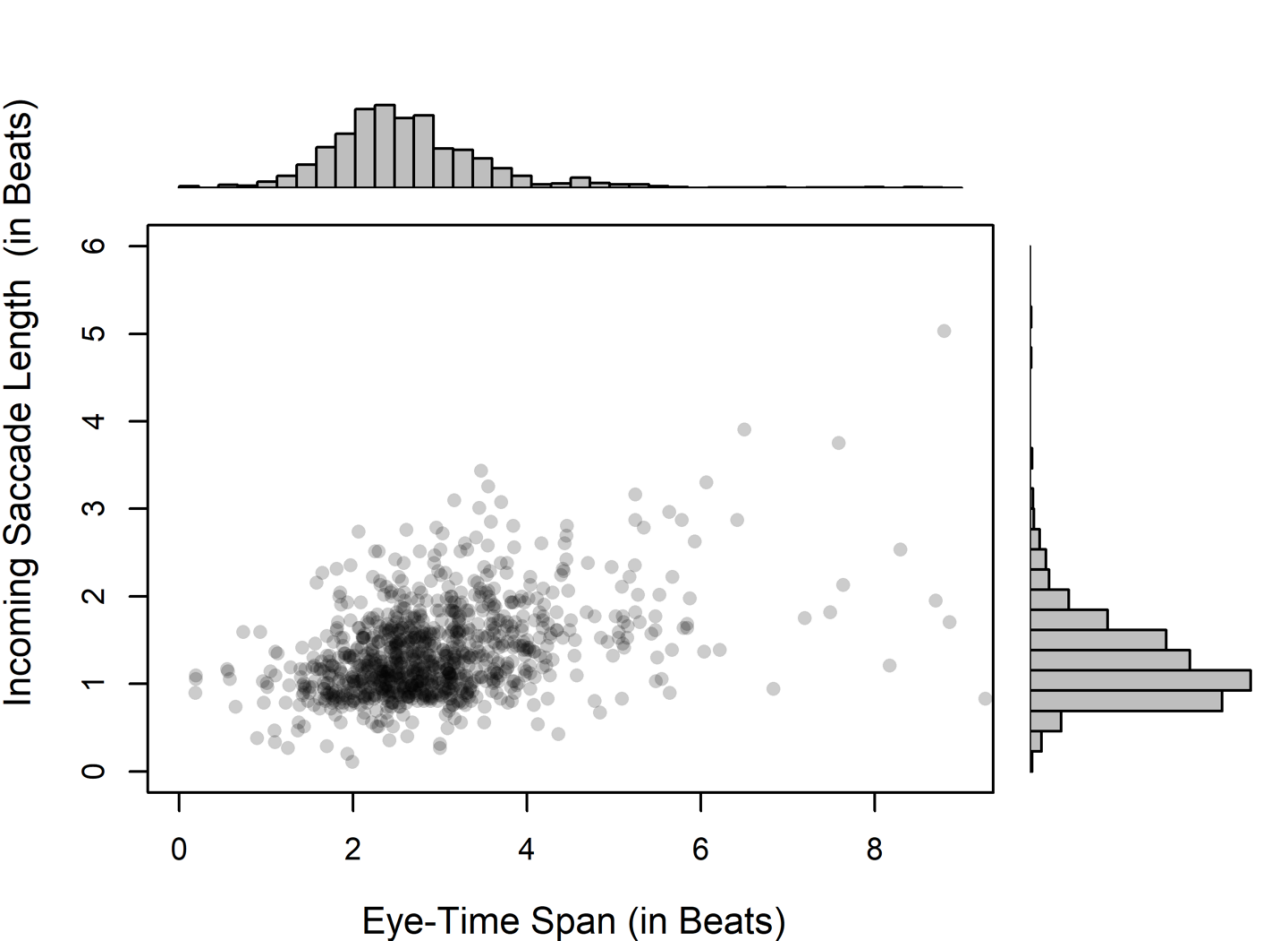
The relationship between incoming saccade length and ETS (with histograms for both variables) at the tempo of 100 bpm in Experiment 2.

As in Exp. 1, we may also take a look at the relationship between ETS and the length of incoming saccades, pooling all participants and conditions together for this analysis. According to Spearman rank correlations, there was a positive correlation between ETS and incoming saccade length both in the tempo of 60 bpm (*ρ*[1,033]= 0.458, *p* < .001), and at 100 bpm (*ρ*[977]= 0.370, *p* < .001). As exemplified in Fig. 11 for the higher tempo, the bulk of the first fixations have been approached through 1–2-beat saccades, resulting in an ETS of roughly 2–4 beats. However, longer ETS measurements tend to correspond to slightly longer incoming saccades.

### Discussion of Experiment 2

In relation to the first study reported above, Experiment 2 was designed to intensify the visual salience and music-structural complexity of the target notes appearing amidst diatonic, stepwise melodies. This was done in order to learn whether increasing the complexity of the target notes would also intensify the attraction effects. In addition, we worked only with highly experienced music readers. Again, the results supported both the Early Attraction Hypothesis and the Distant Attraction Hypothesis, although not directly at the “most complex” note. By interspersing our stepwise melodies with skips of a sixth that landed on chromatically altered notes (involving an accidental sign # or b), we did elicit a local increase in the ETSs, but not at the target notes themselves. Rather, there was a note-specific increase in the ETS for notes preceding the target element by 1–3 beats. Furthermore, incoming saccade length was found to increase for the Skip bars as a whole, but the effect could not be localized at any particular note in the bar—which again shows that the effect was already present even three notes before the “complex” target. 

A reasonable interpretation would be that the addition of accidentals made the target notes visually more salient, causing them to be perceived earlier within the parafoveal region. This would attract a fixation toward the target symbol, often undershooting the target itself. Supposing that the perceptual span would render the target visible from such a landing site (see above), we may further speculate that the target might already be partially processed from this location. Such an assumption cannot be further evaluated with the present data, but it would help explain why there were no attraction effects for the target note itself. Indeed, decoding upcoming notes from the perceptual span is a possibility that makes it uncertain whether “undershooting” the target can at all be understood as an unsuccessful attempt to hit the target. With the possibility to use the perceptual span, the experienced music reader might simply tend to avoid excessive forward saccades to stay in tempo. In the face of a potentially difficult target, a slightly lengthened saccade toward it might suffice to give the extra processing time needed.

Overall, we learn from this experiment that experienced musicians may, indeed, react quite sensitively to upcoming deviant elements. While our target notes were always on the last beat of a 4/4 bar, they nevertheless generated longer-than-average spans to notes located some beats before the target itself. This implies that the sight readers may often have initially reacted to the target element when the metrical time was running as much as six beats (i.e., 1.5 bars) from the target. The reaction might then involve a quick adjustment of the span process by a longer-than-average forward saccade that might not always reach the salient target itself.

## General discussion

While reading and playing music from a notated score, musicians typically direct their eyes somewhat ahead of the notes currently performed. Though several prior studies have examined this phenomenon of “looking ahead,” the part the musical score itself plays in influencing the visual processing has been under-examined (28). In this article, we have addressed the possibility of stimulus-driven local adjustments to the span of looking ahead, suggesting that even small increases in musical complexity might catch the reader’s eyes from farther away than would be the case with simpler material. This Early Attraction Hypothesis requires a new approach to span measurement. Instead of previous measures of the Eye-Hand Span, we argue that local stimulus-driven effects can better be approached via a construct called the Eye-Time Span (ETS), resulting from a Backward Projective Approach to span measurement. 

The ETS minimally involves two basic features. The first of these is that the reader’s first fixation to a target, at the “front end” of the span, will be adopted as the point of departure for the measurement. In comparison to what we have called the Forward Projective Approach—starting at the “back end” of the span—ours has the advantage of being directly oriented toward the musical targets of interest in the score. The other basic feature of the ETS is that the measurements are not taken from the fixation to the “hand” of the performer, but rather to a location in metrical time. In comparison to another previous approach, here called the Single-Item Lag Approach—that measures the temporal distance between fixating a note and playing it—the ETS thus has the advantage of being independent of the musical performer’s interpretive choices and performance failures. In our Backward Projective Approach to span measurement, the location of a fixation in the score can be thought to correspond to a point in the metrical scheme, allowing us to calculate its distance from another such point—namely, from where the metrical time was running at the occurrence of the fixation (see Figs. 1 and 2). In measuring such a distance, we are admittedly relying on an idealized view of the musical score as a one-to-one map of the musical meter, but with appropriately designed stimuli, this strategy allows a more precise grasp of fine, stimulus-driven span adjustments made during the reading process. (The idealization could be relaxed by assigning metrical positions to fixations locally, adjusting conversions from graphical to metrical distance according to the relative widths of bars, or, say, according to the relative distances of first notes appearing on successive beats.) In principle, the ETS could be used as a general measure to be assigned to each fixation, even without indexing the fixations to notational symbols. As our interest was in the musician’s quick reactions to melodic events at the very first encounter, however, we applied it only to first fixations falling on individual AOIs.

In this first fixation oriented paradigm, we sought to address the question of whether and how changes in the relative complexity of notated musical stimuli would affect the sight-readers’ looking-ahead behavior when they are supposed to perform at a regulated tempo. Inspired by the idea of spatial attraction to irregular symbols presented in the parafovea (in text reading: see (9)), we suggested that in temporally controlled music reading, relatively complex upcoming symbols (or symbol relationships) might not only attract next fixations in a spatial sense, but also in a temporal sense—by precipitating the forward saccade. In other words, we supposed that even if simple sight-reading may otherwise proceed by a rather regular succession of saccades from one note to another (15, 55), slightly more complex notated elements or relationships might attract fixations relatively early during the process. This Early Attraction Hypothesis was derived from two simple observations: In music reading, the musical meter and tempo limit the total processing time available, but notated passages may vary in their difficulty for the reader. Supposing that more complex notated events require a larger share of the limited processing time available, a music reader would do well to cultivate quick sensitivity to any upcoming difficulties. While the Early Attraction Hypothesis as such can be tested using the ETS measure, we also noted that the precise interpretation of any such effects would additionally require the analysis of the length of incoming saccades involved. In particular, we further supposed that the early eye-movement reactions to upcoming complexities would take place by relatively long forward saccades toward the difficult elements “popping out” of the parafovea. This was called the Distant Attraction Hypothesis. Neither Early Attraction nor Distant Attraction has previously been empirically reported in studies of music reading. For comparison, it can be noted that, while Distant Attraction has not been attested in text reading (9), Early Attraction would be akin to the “magnet account” that Hyönä and Bertram (14) use to account for so-called inverted parafoveal-on-foveal effects that have been reported in some reading studies (19). If the idea of Early Attraction to parafoveally observed difficulties feels “counterintuitive” in linguistic contexts (14), p. 124, it gains a whole new relevance in the context of temporally constrained music, as explained in the introduction.

We reported two experiments in which amateur (Exp. 1) and professional musicians (Exps. 1, 2) sight-read simple, diatonic melodies in stepwise melodic conditions and in slightly more demanding ones containing skips. For the latter, the melodies were interspersed with larger melodic skips of a fourth or a fifth (Exp. 1), or intervals of a sixth with an accidental (#, b) on the note following the skip (Exp. 2). It was supposed that identifying the note after the skip would require relatively more processing time, and that it would also be visually more salient than the other notes. Two relaxed tempi (60 bpm and 100 bpm) were used in both studies, and both the ETS as well as incoming saccades were analyzed for notes surrounding the skips in errorless keyboard performances.

Our main results were in line with both the Early Attraction Hypothesis and the Distant Attraction Hypothesis. As regards Early Attraction, measurements of ETS in both experiments showed a significant interaction of melodic Condition and melody Note, suggesting that the addition of melodic skips locally increased the amount of looking ahead just as we had supposed. In Exp. 1, the local increase in ETS most clearly appeared on the notes after the melodic skip where it had been expected. In one of the conditions, we also observed heightened values on the note preceding the skip. The latter effect seemed to take over in Exp. 2, in which the music-structural complexity of the skip, as well as the visual saliency of the note following the skip, had been intensified. Here, the ETS was seen to increase on the three notes preceding the skip. In order to interpret these effects, we evaluated the Distant Attraction Hypothesis by examining the lengths of incoming saccades. In Exp. 1, we observed a significant Condition:Note interaction, also apparently due to an increase of incoming saccade lengths right after the skips. In Exp. 2, there was just a highly significant main effect of Condition, showing a lengthening of incoming saccades in the Skip condition, but suggesting, again, that the increase already occurred for several notes preceding the skip. In both of the experiments (and at both of the tempi), we further observed significant positive correlations between the measurements of ETS and incoming saccade length. 

In sum, then, we see that (1) the appearance of even slight melodic complexities in the score may locally increase the amount of looking ahead in sight reading, and that (2) local increases of looking ahead have a lot to do with using extended saccades—both in general, as well as specifically in the case of responding to melodic complexities. As noted, however, with the somewhat longer and more realistic melodic stimuli of Exp. 2 (also involving visually more salient and musically more complex targets), the looking-ahead and saccade effects already occurred on the notes preceding the targets themselves. Such a phenomenon could be interpreted as a saccadic range error (44) in which saccades, shot to the parafovea for quickly “checking out” upcoming difficulties, may undershoot their targets (17). However, leaving it at that might be to misrepresent the situation in which not only the upcoming target, but all of the notes in between have to be taken care of in the performance. A safer interpretation would be that upcoming, salient difficulties simply tend to increase saccade length and thus expedite the scanning process as a whole. Instead of necessarily aiming to “hit the target,” the reader, observing an upcoming irregularity or difficulty, will simply tend to proceed faster toward it. As noted in the introduction, a perceptual span extending 2–4 beats to the right from a fixation (32, 34, 45) might allow the reader to register the target even from a prior position. However, no such supposition is required to make sense of the basic idea that precipitating the whole process by one or more longer saccades may help the reader get faster to the parafoveally observed irregularities. While the present data cannot reveal the extent to which the sight-reading performances would fail in the absence of such saccadic precautionary measures, it is notable that in our data set, these phenomena were observed as a mark of successful (i.e. errorless) sight-reading performances by highly experienced music readers.

These results may now be compared to ones from two previous studies in which the effects of relative music-structural complexity on the Eye-Hand Span have been addressed in temporally controlled performances. First, in a simple children’s song context, Penttinen et al. (4) observed that musicians’ Eye-Hand Spans, when measured at beat onsets (as “Eye-Beat Spans”), were shorter for beats involving eighth notes than for beats involving quarter notes. Second, Rosemann et al. (33) had pianists perform the accompaniment score to a Bach flute sonata, and found that bars rated by the authors as “difficult” received a shorter Eye-Hand Span than was the case for bars rated as “easy.” Both of these studies thus suggested that musical complexity would reduce the spans, instead of increasing them. The reason for this, of course, is that both of the studies applied Forward Projective measures: Longer spans were reported for “difficult” areas, but these were spans originating from—and not targeting—the areas in question (see also (33), supplementary material). In both of these previous studies, the reduced spans measured at complex areas may reflect the fact that the reader has spent more time on these events than on others, and hence the scanning of the next events, after the complex ones, has been delayed. These would be just after effects of complexity, and not effects of spotting the complexities in the first place. When spans are grouped by their point of origin and not by their targets, the whole measurement process may fail to show what we take to be the most important relationship between musical complexity and looking ahead—namely, the way in which upcoming complexities instigate early oculomotor responses.

Even if the ETS thus seems a more adequate tool than previous Eye-Hand Span measures to record local, stimulus-driven changes in the musician’s spans, the present results also suggest that it should not be taken as a direct measure of element salience. This is because even if visually salient and/or musically complex symbols yield a local increase in ETS, the longer spans might not be targeted exactly at the elements of interest. By the same token, the most immediate complexity effects of a given score area cannot be found by the Single-Item Lag approach either. The fixation data that represents the most immediate reaction to a given note symbol might not at all be located right at that symbol. Answering questions concerning stimulus-driven effects on looking ahead thus requires taking into account the first fixations to a broader visual area extending backward in the score from the actual elements of interest. What this implies is that future studies of eye movements in music reading should strive to understand phenomena such as looking ahead in terms of broader models of the sight-reading process.

In one of the rare models explicitly proposed for saccadic control in music reading, Kinsler and Carpenter (55) overlooked the possibility that stimulus features such as points of relative complexity might affect saccadic programming. Instead, they supposed that a neurally encoded image of local notational symbols is “scanned internally by a processor which also triggers the saccadic controller when there is no more material that can be processed” (55), p. 1456. Accordingly, the authors suggested that saccades “[i] are not initiated at times that are particularly significant from the point of view of the music and its performance, [ii] nor are they directed consistently in relation to visual elements on the page” (55), p. 1454. There is reason to doubt Kinsler and Carpenter’s view, however, as their model was based on rather informal observations concerning simple rhythmic tapping tasks (with only four experimental participants, apparently including themselves), and their notated stimuli did not involve any particular symbols that could have saliently popped out from the parafovea due to their intrinsic or relational complexity. In light of the present results, Kinsler and Carpenter’s claims grossly overstate the fact that neither the individual launch times nor the precise landing positions for saccades can be deterministically predicted from the notated stimulus. Even granting flexibility in the individual saccadic processes, we have seen that in relation to the course of metrical time, (i) saccades tend to be initiated earlier in response to upcoming complex symbols (or symbol relationships), and that (ii) such saccades are drawn closer to the symbols in question. These phenomena of Early Attraction and Distant Attraction seem to take place within flexible, individually variable saccadic processes which nevertheless—on a group level—respond in largely predictable ways to slight changes of structural complexity in the musical notation. In this sense, at least, music reading is much more a “genuine species of music perception” (46), p. 235, than Kinsler and Carpenter’s model would have it. Crafting a more appropriate model of saccadic control in sight reading lies beyond the scope of this article, but we think we have been able to show that such a model would have to take into account both the temporal restrictions placed on music reading as well as the bottom-up influence of the notated stimulus on the reading process.

Our study shows some shortcomings that could be taken into account in planning future research. On the technical side, while the eye-tracking and stimulus presentation were synchronized by the eye-tracker, and while our approach allowed disregarding the performer’s actions on the keyboard (beyond a separate check for correctness), we still had to synchronize the eye-tracking data with the external metronome (provided by the sequencer software recording the performance). Here, we relied on identifying beat onsets through a set of reference points given by the experimenter’s actions, as she had changed the screen images on the eye-tracker in sync with the heard metronome. Even if the experimenter could time her actions with the metronome (see above), the procedure represents a source of potential error which could be obviated by the use of automated procedures in future work. Nevertheless, the variability in the researcher’s actions was measured to be on the scale of some milliseconds, while the significant effects found for the ETS, for instance, were large enough (typically hundreds of milliseconds) to not be significantly affected by it.

Another, more theoretical issue that would merit further attention in future work has to do with the notion of musical complexity governing our stimulus design. Two different aspects are relevant to mention here. First, it should be clear that we have only used the notion of complexity as a heuristic way of approaching what is a much more intricate music-theoretical and music-psychological issue. Even our simple melodies involved an interplay of two very different kinds of sources for melodic complexity: chromaticism (i.e. departure from diatonic scales by way of using accidentals), and interval size. Furthermore, in both cases, there might be several possibilities for defining complexity. Our account has been in terms of expected processing load. Hence, we suggested that larger melodic intervals involve greater cognitive difficulties than smaller stepwise ones which might be more easily decoded as up/down commands on an underlying learned scale. One might also argue for the greater difficulty of larger intervals on the basis of their smaller frequency in melodic corpora (56). The problem, however, is that musical pitch is a multidimensional phenomenon and that non-stepwise intervals, too, might be deemed structurally simple, as is the case for fourths and fifths which not only can be used as generators to produce the whole Western tone system (in the so-called circle of fifths), but also emerge in tonal perception as cognitively stable relationships (57). In this light, any music-structural complexity attached to these intervals in our Experiment 1 would not be about inherent structural complexity of the relationships themselves, but due to the increase in local melodic variability and—to repeat our previous point—a greater need to identify the following note as such.

As regards the topic of early attraction, an even more crucial aspect concerning music-structural complexity is the close association between such complexity and visual salience in written music. The roots of this association are in the very system of musical notation itself which can represent simple diatonic (e.g., major or minor) melodies without accidental symbols, whereas more complex chromatic pitch structures would also require such added visual markings. In future studies, more attention could be paid to this aspect—for instance, by involving conditions in which simple diatonic melodies are written with accidentals, so as to visually assimilate them with less familiar music-structural materials (cf. 1). Indeed, magnetic attraction is less puzzling in our study than in text reading just because of the visual salience aspect involved, and thus it might be of interest to see if our results can be extended to contexts where the relative visual salience of musically complex events is attenuated. Our guess would be that they cannot be so extended. Musical common sense suggests explaining our present results by the musicians’ learned skills of navigating in well-notated scores in which simple things are notated in visually simple ways, if possible, and where visually salient features tend to signal musical complexities, as well. In this light, the phenomenon of early attraction in music reading would be first and foremost a matter of reacting to visually salient cues regarding locations of potential musical interest or challenge.

In this article, we have shown that the music reader’s extent of looking ahead of metrical time can be sensitive to local variations in musical structure on a note-to-note level. In closing, it is relevant to emphasize that despite advances in measuring the phenomenon, the amount of looking ahead probably cannot be determined solely on the basis of stimulus properties. This is because early attraction, as noted above, may be based on a useful habit of reacting to any salient points of interest—whether or not the extra time thereby gained will actually be needed or not. Indeed, the temporal buffer obtained by looking ahead may be used for any and all of the purposes that the reader might need. While our present methods do not allow an analysis of how much of the temporal buffer is used for decoding the upcoming symbol and how much of it goes to preparing for motor execution, this is most likely to vary between individuals according to their music-theoretical knowledge and instrument-specific skills. Moreover, in real music-reading contexts, the temporal buffer provided by looking ahead can be used for practical purposes that we have not even touched upon, such as collecting information from written expressive markings, managing page turns, or glancing for synchronizing cues from fellow musicians or a conductor, and more. In this sense, the phenomenon of early attraction presents just one—although an important—aspect of looking ahead in music reading.

### Ethics and Conflict of Interest

The authors declare that the contents of the article are in agreement with the ethics described in http://biblio.unibe.ch/portale/elibrary/BOP/jemr/ethics.html and that there is no conflict of interest regarding the publication of this paper.

### cknowledgements

This research was supported by the grant 275929 from the Academy of Finland, and by the Turku Institute for Advanced Studies. We wish to thank Lauren Fink, Jukka Hyönä, Elke B. Lange, Jochen Laubrock, Antti Penttinen, and two anonymous reviewers for critical comments, Nina Loimusalo and Markku Pöyhönen for assistance, Irmeli Matilainen for eyes and hands, as well as the participants of the two studies for their time and effort.

## Appendix

**Appendix 1.** Parameter estimates (in beats) for Eye-Time Span, Experiment 1.

	Estimate	Std.err	Wald	Pr(>|W|)
(Intercept)	0.5429	0.0296	337.512	< .001 ***
expertisePM	-0.0813	0.0298	7.464	.006 **
tempo100	-0.0597	0.0127	22.170	< .001 ***
conditionB	-0.0029	0.0131	0.048	.827
conditionM	0.0106	0.0125	0.719	.396
note4bar2	0.0268	0.0145	3.417	.065
note1bar3	-0.0120	0.0190	0.397	.529
note2bar3	0.0055	0.0155	0.123	.726
note3bar3	-0.0010	0.0213	0.002	.963
note4bar3	0.0267	0.0239	1.256	.263
conditionB:note4bar2	-0.0481	0.0144	11.222	<.001 ***
conditionM:note4bar2	-0.0127	0.0157	0.655	.418
conditionB:note1bar3	-0.0229	0.0185	1.528	.216
conditionM:note1bar3	0.0098	0.0227	0.186	.666
conditionB:note2bar3	-0.0036	0.0200	0.032	.859
conditionM:note2bar3	-0.0351	0.0177	3.916	.048 *
conditionB:note3bar3	0.0058	0.0271	0.046	.831
conditionM:note3bar3	-0.0596	0.0218	7.480	.006 **
conditionB:note4bar3	-0.0098	0.0297	0.109	.741
conditionM:note4bar3	-0.0817	0.0319	6.552	.010 *

Estimated Scale Parameters:	Estimate	Std.err		
(Intercept)	0.165	0.0201		

Estimated Correlation Parameters:	Estimate	Std.err		
alpha	0.259	0.0488		

Number of clusters: 37 Maximum cluster size: 70				

*Signif. codes*: * *p* < .05; ** *p* < .01; *** *p* < .001.*Abbreviations*: expertisePM = Performance major group, tempo100 = tempo 100 bpm, conditionB = Bar line condition, conditionM = Mid-bar condition, note4bar2 = note 4 of bar 2, note1bar3 = note 1 of bar 3. *Reference level used*: Education major group, tempo 60 bpm, Stepwise-condition, note 3 of bar 2.

**Appendix 2.** Parameter estimates (in beats) for Incoming Saccade Length, Experiment 1.

	Estimate	Std.err	Wald	Pr(>|W|)
(Intercept)	0.9283	0.0343	732.686	< .001 ***
expertisePM	-0.0847	0.0299	8.031	.005 **
tempo100	-0.0006	0.0218	0.001	.980
conditionB	-0.0095	0.0365	0.067	.796
conditionM	0.0197	0.0396	0.246	.620
note4bar2	0.0704	0.0458	2.359	.125
note1bar3	-0.0290	0.0336	0.745	.388
note2bar3	-0.0291	0.0606	0.231	.631
note3bar3	-0.0595	0.0547	1.184	.277
note4bar3	0.0398	0.0660	0.364	.546
conditionB:note4bar2	-0.0319	0.0629	0.257	.612
conditionM:note4bar2	-0.0135	0.0660	0.042	.838
conditionB:note1bar3	-0.0704	0.0389	3.279	.070
conditionM:note1bar3	-0.0278	0.0386	0.519	.471
conditionB:note2bar3	0.0472	0.0831	0.323	.570
conditionM:note2bar3	-0.0616	0.0642	0.920	.337
conditionB:note3bar3	0.0645	0.0637	1.026	.311
conditionM:note3bar3	-0.0390	0.0702	0.309	.578
conditionB:note4bar3	-0.0219	0.0755	0.084	.772
conditionM:note4bar3	-0.0927	0.0924	1.006	.316

Estimated Scale Parameters:	Estimate	Std.err		
(Intercept)	0.224	0.0300		

Estimated Correlation Parameters:	Estimate	Std.err		
alpha	0.0226	0.0095		

Number of clusters: 37 Maximum cluster size: 48				

*Signif. codes*: * *p* < .05; ** *p* < .01; *** *p* < .001*. Abbreviations*: as in Appendix 1. *Reference level used*: as in Appendix 1.

**Appendix 3.** Parameter estimates (in beats) for Eye-Time Span, Experiment 2.

	Estimate	Std.err	Wald	Pr(>|W|)
(Intercept)	0.3822	0.0177	463.840	< .001 ***
tempo100	-0.0113	0.0109	1.085	.298
conditionSkip	-0.0532	0.0083	41.076	< .001 ***
note2	-0.0147	0.0113	1.698	.193
note3	-0.0125	0.0076	2.697	.101
note4	-0.0185	0.0133	1.924	.165
conditionSkip:note2	-0.0116	0.0115	1.021	.312
conditionSkip:note3	-0.0008	0.0143	0.003	.956
conditionSkip:note4	0.0451	0.0176	6.592	.010 *

Estimated Scale Parameters:	Estimate	Std.err		
(Intercept)	0.1912	0.0382		

Estimated Correlation Parameters:	Estimate	Std.err		
alpha	0.1573	0.0636		

Number of clusters: 14 Maximum cluster size: 160				

*Signif. codes*: * *p* < .05; ** *p* < .01; *** *p* < .001.*Abbreviations*: tempo100 = tempo 100 bpm, conditionSkip = Skip condition, note2 = note 2 of the target bar.*Reference level used*: tempo 60 bpm, Stepwise-condition, note 1 of the target bar.

**Appendix 4.** Parameter estimates (in beats) for Incoming Saccade Length, Experiment 2

	Estimate	Std.err	Wald	Pr(>|W|)
(Intercept)	0.6862	0.0238	832.17	< .001 ***
tempo100	0.0224	0.0180	1.56	.212
conditionSkip	-0.0566	0.0129	19.14	< .001 ***
note2	0.0619	0.0280	4.90	.027 *
note3	0.1074	0.0241	19.80	< .001 ***
note4	0.0541	0.0419	1.67	.197
conditionSkip:note2	-0.0620	0.0312	3.95	.047 *
conditionSkip:note3	-0.0202	0.0314	0.41	.521
conditionSkip:note4	0.0512	0.0536	0.91	.340

Estimated Scale Parameters:	Estimate	Std.err		
(Intercept)	0.1970	0.0265		

Estimated Correlation Parameters:	Estimate	Std.err		
alpha	0.0543	0.0168		

Number of clusters: 14 Maximum cluster size: 160				

*Signif. codes*: * *p* < .05; ** *p* < .01; *** *p* < .001. *Abbreviations* as in Appendix 3. *Reference level used*: as in Appendix 3.
